# Short-Term Pre-Harvest UV-B Supplement Enhances the Polyphenol Content and Antioxidant Capacity of *Ocimum basilicum* Leaves during Storage

**DOI:** 10.3390/plants9060797

**Published:** 2020-06-25

**Authors:** Luana Beatriz dos S. Nascimento, Cecilia Brunetti, Giovanni Agati, Clara Lo Iacono, Cassandra Detti, Edgardo Giordani, Francesco Ferrini, Antonella Gori

**Affiliations:** 1 University of Florence, Department of Agriculture, Food, Environment and Forestry (DAGRI), Section Woody Plants, CAP. 50019 Sesto Fiorentino (Florence), Italy; lulibia.17@gmail.com (L.B.d.S.N.); clara.loiacono@stud.unifi.it (C.L.I.); cassandra.detti@unifi.it (C.D.); edgardo.giordani@unifi.it (E.G.); francesco.ferrini@unifi.it (F.F.); antonella.gori@unifi.it (A.G.); 2 National Research Council of Italy, Institute for Sustainable Plant Protection (IPSP), CAP. 50019 Sesto Fiorentino (Florence), Italy; 3 National Research Council of Italy, Institute of Applied Physics (IFAC), CAP. 50019 Sesto Fiorentino (Florence), Italy; g.agati@ifac.cnr.it

**Keywords:** carotenoids, chlorophyll, DPPH, Dualex^®^ device, HPLC-DAD, hydroxycinnamic acid derivatives (HCAs), *Ocimum basilicum*

## Abstract

*Ocimum basilicum* (basil) leaves are rich in polyphenols, conferring them a high antioxidant activity. The application of UV-B can be used to maintain the post-harvest nutraceutical quality of basil leaves. We aimed to investigate the effects of pre-harvest UV-B application on polyphenolic and pigment contents, antioxidant capacity, and the visual quality of basil stored leaves. We also evaluated the applicability of the non-invasive Dualex^®^ for monitoring the accumulation of leaf epidermal phenolics (Flav Index). After exposing plants to white light (control) and to supplemental UV-B radiation for 4 d, the leaves were harvested and stored for 7d (T_S7_). The UV-B leaves showed both a higher phenolic content and antioxidant capacity than the controls at T_S7_. In addition, the correlations between the Flav Index and phenolic content demonstrated that Dualex^®^ can reliably assess the content of epidermal phenolics, thus confirming its promising utilization as a non-destructive method for monitoring the phytochemical quality of *O. basilicum* leaves. In conclusion, a pre-harvesting UV-B application may be a tool for enhancing the content of polyphenols and the antioxidant potential of basil stored leaves without detrimental effects on their visual quality. These results are important considering the nutraceutical value of this plant and its wide commercial distribution.

## 1. Introduction

As photosynthetic sessile organisms, plants are naturally exposed to harmful UV-B radiation (280–320 nm) and have developed several morphophysiological and biochemical adaptations for dealing with their possible damages [1]. Despite the fact that many studies have focused on the effects of this radiation on plants, the application of UV-B as an elicitor to increase the production of secondary metabolites is currently growing, especially under controlled conditions [2,3]. Indeed, many species have shown an increase in secondary metabolites content, mainly in phenolic compounds, when exposed to UV-B radiation [1,2,4].

Polyphenols are largely distributed in the plant kingdom and play important roles in growth, development and protection against several biotic and abiotic stresses. Flavonoids and phenylpropanoids (hydroxycinnamic acid derivatives—HCAs), in particular, offer protection against UV-B, acting as both radiation screeners and ROS (reactive oxygen species) scavengers [1,2]. These compounds are located in the vacuole of epidermal and subepidermal cells, decreasing the UV-B penetration into the mesophyll [1,5]. Besides this, they are also found in the mesophyll cells, where they perform an antioxidant function [5]. 

Polyphenols are also known as human health-promoting compounds thanks to their biological activities, such as anti-inflammatory, antioxidant, antitumor, and antimicrobial [6]. The content of these compounds in consumable leafy vegetables improves their nutraceutical value and can be increased under controlled abiotic stress conditions [7,8]. In this sense, UV-B radiation can be easily applied during the cultivation and/or the post-harvest process of plant-derived foods to enhance their quality [4]. 

*Ocimum basilicum* L. (cv “Genovese”, a cultivar protected by the European Union with the Denominazione di Origine Protetta certification), or sweet basil, is a highly valued horticultural crop that is widely consumed around the world, especially in Mediterranean and South East Asian countries. The fresh or dried leaves of this species are commonly used as a spice [9,10]. Basil leaves are rich in essential oils and phenolic compounds (phenolic acids, caffeic acid derivatives, and flavonoids), contributing to their high antioxidant activity [10,11,12]. Despite the presence of these compounds, the quality of basil leaves can decrease during transportation and storage, depending on environmental conditions such as temperature and air humidity [10,13]. 

Some studies have reported the effect of UV-B radiation on the phenolic content of basil leaves [3,11,12]. UV-B radiation applied for long periods in a discontinuous manner has been shown to increase the total phenol and total flavonoid contents of basil leaves, as well as the production of some specific compounds (such as cinnamic acid and luteolin) [11,12]. In particular, phenolic production triggered by UV-B and its composition may vary according to the time of exposure. For example, Ghasemzadeh et al. (2016) [11] found that while an increase in phenolic acids occurred with an 8 h of UV-B exposure, higher concentrations of cinnamic acid derivatives were detected when the plants were irradiated for 6 h.

However, the effect of UV-B radiation exposure on phenolic composition and antioxidant activity and its relationship with the postharvest quality of basil leaves have not been studied yet. In addition, to date non-destructive methods to check the phenolic content in response to UV-B exposure in the leaves of basil have not been applied. In this context, non-destructive techniques, such as the ones based on the optical properties of flavonols and on chlorophyll fluorescence (including the Dualex^®^ instrument), could be considered promising commercial and industrial tools to easily monitor the changes in the epidermal flavonol content of leaves exposed to UV-B [14].

The aim of this study is to evaluate the changes in the phenolic content and composition, antioxidant capacity, and visual quality of stored leaves by the pre-harvest exposure of *O. basilicum* potted plants to supplemental UV-B radiation. We hypothesize that UV-B can be applied during the cultivation of *O. basilicumin* order to increase the leaf polyphenolic content and consequently the antioxidant potential, thus enhancing the postharvest phytochemical and visual quality of the stored leaves. In addition, this study aims at assessing the applicability of the Dualex^®^ instrument as a non-destructive tool to monitor the leaf polyphenolic and chlorophyll content in basil leaves.

## 2. Results

### 2.1. Phytochemical Quality of O. basilicum Leaves: Non-Destructive and Destructive Analyses 

**Figure 1:**
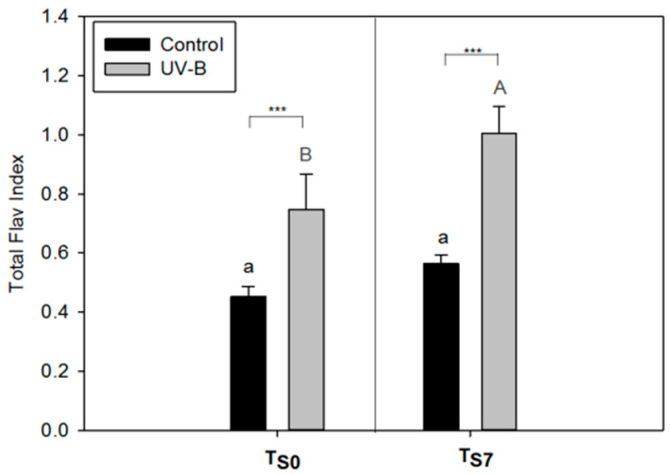
Total Flav Index of leaves of *O. basilicum* plants cultivated under white light (control—black bars) and supplemental UV-B radiation (UV-B—gray bars) at the end of the light treatment (before storage, T_S0_) and after seven days of storage (T_S7_). Mean ± SD (*n* = 3). Letters show the comparison between different storage times (T_S0_ or T_S7_) under the same light conditions (uppercase gray letters—UV-B treatment; lowercase black letters—control). Different letters indicate significant differences between the values (*p* < 0.05), while asterisks show the significant difference between light treatments at the same time of storage (*** *p* ≤ 0.001); ns = not significant.

The UV-B leaves showed significantly higher values of the Total Flav Index than the control leaves at both sampling times, T_S0_ and T_S7_ (Figure 1). Comparing the Flav Index of each leaf side, this parameter significantly increased in UV-B-treated leaves at T_S0_ only on the adaxial epidermis (Flav Index adaxial) (Supplementary Figure S1). By contrast, a significant increment of the Flav Index in UV-B-treated leaves was observed on both the adaxial and abaxial epidermis after 7 d of storage (T_S7_) (Supplementary Figure S1).

Considering the Flav Index results between the different storage moments (T_S0_ vs. T_S7_) within the same light treatment, the UV-B pre-treated leaves showed a higher epidermal phenolic content after the storage (at T_S7_) than before it (at T_S0_) (for Flav Index adaxial, abaxial, and total, Figure 1 and Supplementary Figure S1). On the other hand, the differences in the Flav Index between T_S0_ and T_S7_ in the control leaves were observed only on the abaxial epidermis (Supplementary Figure S1b).

**Figure 2:**
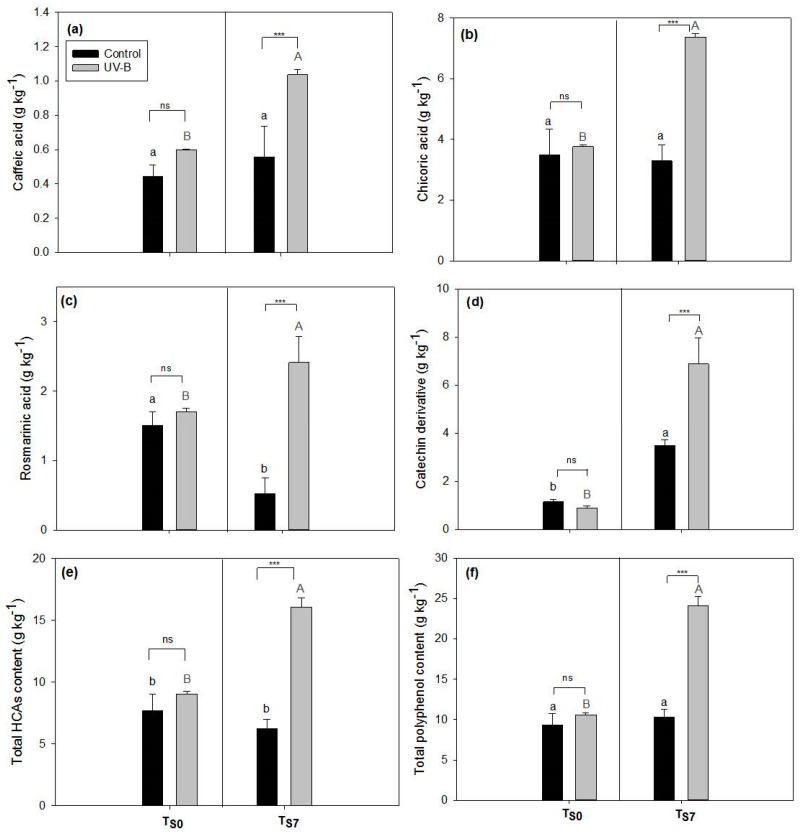
Polyphenolic content ((**a**–**f**), in g kg^−1^ of dry weight) of leaves of *O. basilicum* plants cultivated under white light (control—black bars) and supplemental UV-B radiation (UV-B—gray bars) at the end of the light treatment (before storage, T_S0_) and after seven days of storage (T_S7_). Mean ± SD (*n* = 3). (**a**) Caffeic acid content, (**b**) chlorogenic acid content, (**c**) rosmarinic acid content, (**d**) catechin derivative content, (**e**) total hydroxycinnamic acid derivatives content (HCAs), (**f**) total polyphenol content. Letters show the comparison between different storage times (T_S0_ or T_S7_) under the same light conditions (uppercase gray letters—UV-B treatment; lowercase black letters—control). Different letters indicate significant differences between the values (*p* < 0.05), while asterisks show a significant difference between light treatments at the same time of storage (*** *p* ≤ 0.001); ns = not significant.

Extracts of leaves collected at both storage times (T_S0_ and T_S7_) and from both light treatments showed similar polyphenolic profiles (Supplementary Figure S2). They were shown to be rich in hydroxycinnamic acid derivatives (HCAs), and among these, chicoric (peak 8), rosmarinic (peak 10) and caffeic acids (peak 5) were the most abundant. In addition, a catechin derivative (peak 12) was detected, especially in the extracts of leaves pre-treated with UV-B and subsequently submitted to storage (T_S7_). Despite their similar polyphenolic profiles, the amounts of these most abundant compounds in the extracts were different according to both the light treatment (control vs. UV-B) and the sampling time (T_S0_ vs. T_S7_) (Figure 2). 

The content of polyphenols in the extracts of leaves at T_S0_ (Figure 2a–f) did not show differences according to the light treatments (control vs. UV-B) for any of the compounds detected by high performance liquid chromatography, coupled to a diode array detector (HPLC-DAD). On the other hand, the content of all the different polyphenols after 7 d of storage (at T_S7_) was shown to be significantly higher in the leaves pre-harvest exposed to UV-B radiation compared to the control (T_S7_, Figure 2a–f). Comparing the results between storage times (T_S0_ vs. T_S7_) within the same light treatment, the control leaves showed significant differences only in the content of rosmarinic acid (decrease of around 65%, Figure 2c) and the catechin derivative (increase of around 67%, Figure 2d). On the other hand, a marked increase in the content of all the polyphenolic classes was observed in the UV-B pre-treated leaves after 7 d of storage compared to T_S0_ (Figure 2a–f). For the total polyphenols content, an increase of around 56% was observed.

**Figure 3:**
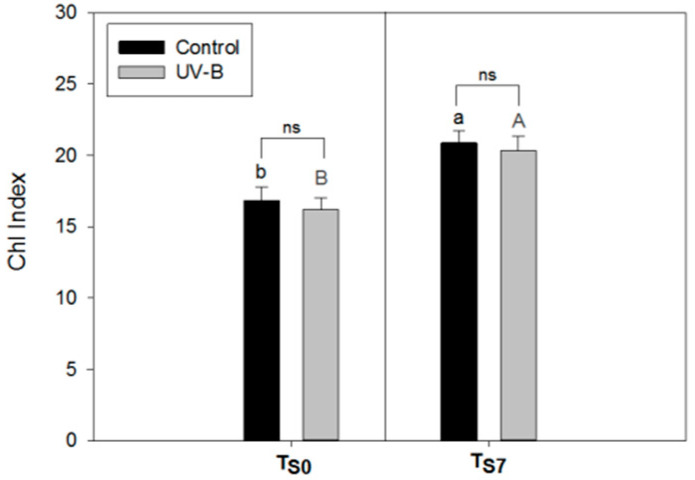
Chl Index of leaves of *O. basilicum* plants cultivated under white light (control—black bars) and supplemental UV-B radiation (UV-B—gray bars) extracted immediately at the end of the light treatment (before storage T_S0_) or after seven days of storage (T_S7_). Mean ± SD (*n* = 3). Letters show the comparison between different storage times (T_S0_ or T_S7_) under the same light conditions (uppercase gray letters—UV-B treatment; lowercase black letters—control). Different letters indicate significant differences between the values (*p* < 0.05), while asterisks show the significant differences between light treatments at the same time of storage; ns = not significant.

An increase in the Chl Index values in both light treatments (control and UV-B) was observed after the storage time (Figure 3). For this parameter, no differences between the light treatments were observed at both T_S0_ and T_S7_.

**Figure 4:**
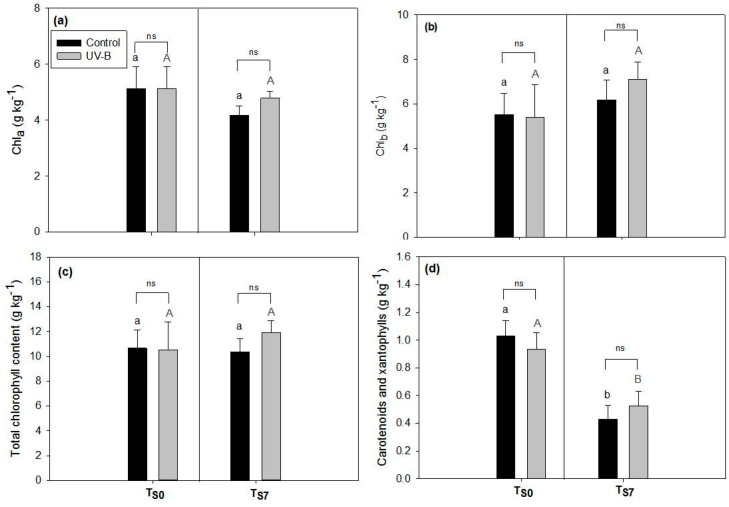
Photosynthetic pigment content ((**a**–**c**)—chlorophylls *a*, *b*, and total, respectively; (**d**)—carotenoids, in g kg^−1^ of dry weight) of leaves of *O. basilicum* plants cultivated under white light (control—black bars) and supplemental UV-B radiation (UV-B—gray bars) at the end of the light treatment (before storage T_S0_) or after seven days of storage (T_S7_). Mean ± SD (*n* = 3). Letters show the comparison between different storage times (T_S0_ or T_S7_) under the same light conditions (uppercase gray letters—UV-B treatment; lowercase black letters—control). Different letters indicate significant differences between the values (*p* < 0.05), while asterisks show significant differences between light treatments at the same time of storage; ns = not significant.

The content of chlorophylls (*a*, *b*, and total) did not change according to either the time of storage (T_S0_ vs. T_S7_) or the light treatment (control vs. UV-B) (*p* > 0.05, Figure 4a–c). The content of carotenoids (Figure 4d) significantly decreased after seven days of storage (*p* < 0.001, T_S0_ vs. T_S7_) in both the control and UV-B leaves (a decrease of around 58% in the control leaves and of 44% in the UV-B leaves, respectively), without differences between them (*p* = 0.99).

### 2.2. Antioxidant Capacity of Leaf Extracts: Effects of Light Treatments and Storage

At T_S0_, no differences in the EC_50_ values between the control and UV-B leaf-extracts were observed (Table 1), while the EC_50_ of extracts of UV-B pre-harvest-exposed leaves after 7 d of storage (at T_S7_) were shown to be around five-fold lower than the controls. 

The correlation analysis between the EC_50_ values and the content of the different polyphenols showed to be significant for rosmarinic and chicoric acids, as well as for the total content of HCAs and total polyphenols (Table 2).

### 2.3. Visual Quality of O. basilicum Leaves: % FWLR and Colorimetric Analysis

**Figure 5:**
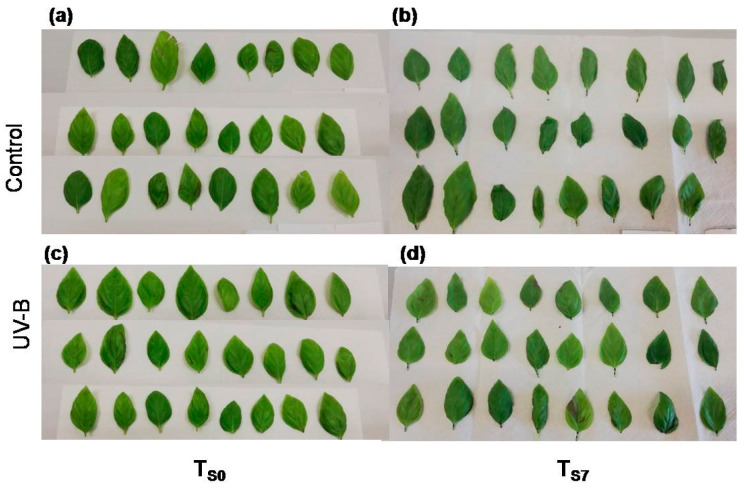
Pictures of *O. basilicum* leaves from the control (**a**,**b**) and UV-B treatments (**c**,**d**) at T_S0_ (**a**,**c**) and at T_S7_ (**b**,**d**).

At T_S0_, the leaves of *O. basilicum* did not differ in appearance between the light treatments (control or UV-B—Figure 5a,c, respectively). Indeed, the UV-B treatment did not induce any visible necrosis, darkness, or visible injuries (Figure 5c) when compared to the control leaves (Figure 5a). However, after seven days of storage (at T_S7_—Figure 5b,d), the leaves of both light treatments appeared to be more wilted than at T_S0_ (Figure 5a,c).

The leaf fresh weight decreased significantly during the storage time in both the light treatments (Table 3). The lowest fresh weight (FW) values (around 35% of the FWLR—fresh weight loss rate) were achieved after seven days of storage, while no significant differences were observed between the two light treatments.

The storage of leaves caused an increase in the *L* values (Table 4). In addition, the leaves from plants pre-exposed to UV-B showed lower *L* values than the controls. The *a* values increased at T_S7_, without differences between the light treatments. The results of the ∆E (around 3.0) showed that the storage time induced significant changes in the color of the leaves from both light treatments. No significant differences were observed for this value according to the light treatment.

### 2.4. Applicability of the Non-Destructive Dualex^®^ Device

The correlation between the content of the main polyphenolic compounds and the Total Flav Index (measured by Dualex^®^) was significant (*p* < 0.001) and high (*r* ≥ 0.85) for each identified polyphenol (data not shown), as well as for the total amount of HCAs and total polyphenolic content (Table 5). The correlation between the Chl Index and the total chlorophyll content was not significant (*p* = 0.86). 

## 3. Discussion

### 3.1. Effect of UV-B Treatment on Post-Harvest Phytochemical Quality of O. basilicum Leaves

In recent years, the consumption of fresh-cut leafy vegetables and spices has gained popularity, and this market segment has shown a greatest economical progression in the food industry [15]. Therefore, the efforts and investments into the post-harvest quality and maintenance of these products have likewise increased. Since fresh-cut products have a faster and more pronounced deterioration than processed food, the losses for industries represent an important burden [15]. As such, the development of eco-friendly techniques to preserve or enhance the postharvest phytochemical quality of these products is significantly important [7,15]. Indeed, some bioactive compounds, in particular polyphenols and carotenoids, are essential for maintaining the quality of leafy vegetables and enhancing their nutritional value, especially thanks to their antioxidant activity [8]. In this sense, the application of some abiotic stresses as elicitors for the biosynthesis of these compounds, such as the application of UV-B radiation, are regarded as promising technologies for this purpose [7,15]. 

In our experiment, we observed that the UV-B treatment applied during the cultivation of basil triggered an increase in the Flav Index in *O. basilicum* leaves at both T_S0_ and T_S7_, especially on the leaf adaxial side (Figure 1, Supplementary Figure S1). The increase in the UV-filter compounds in epidermal and subepidermal surface layers is a common response to UV-B exposure [1,16]. These protective compounds, including flavonoids and HCAs, are generally accumulated in the vacuoles of epidermal cells and act in decreasing the transmittance of UV wavelengths to the mesophyll, protecting the deeper photosynthetic layers of the leaf [1,17]. The increase the in Flav Index in response to UV-B exposure was also observed in some previous experiments with *Centella asiatica* [18] and *Betula pendula* [19].

Our results suggest that the increase in the epidermal leaf phenolics might be “the first step” in defense against UV-B, since it was observed immediately after the end of the UV-B exposure (at T_S0_), even without an increase in the leaf polyphenolic content measured by HPLC-DAD at this time (T_S0_, Figure 2). Indeed, although the UVB-mediated accumulation of phenolics may occur in different leaf tissues by the action of the UVR-8 photoreceptor, this happens in a tissue-autonomous way and the epidermis shows to be the primary site of UV-B perception, followed by the subepidermal and mesophyll cells [20]. Thus, changes in the UV-B-absorbing phenolics in leaf surface tissues could be considered as the primary mechanism for acclimation to this type of stress. This is also in agreement with a previous work showing a low UV-B transmittance (less than 10%) into tissues below the epidermis [17,21]. 

In addition, the UV-triggered accumulation of the epidermal phenolics of *O. basilicum* leaves (Flav Index) showed to be even higher at the end of the storage (at T_S7_) (Figure 1). This increment might indicate a constitutive UV-defensive answer, since epidermal phenolics were not degraded with the storage (Figure 1, Supplementary Figure S1). Similar results of high Flav Index values after the interruption of UV-B exposure were previously observed in *Capsicum annuum* [22]. However, we cannot exclude that the observed increment in the Flav Index at T_S7_ might be also due to the low temperature of the storage. In fact, protective responses against UV-B radiation can be observed as a possible pre-acclimation response to further abiotic stresses caused by a new exposure to UV radiations or to other factors [22,23,24,25]. Indeed, the increment in phenolic compounds caused by low temperatures is already described in the literature [26] and can be attributed to the cold-induced activity of phenylalanine ammoniumlyase (PAL) and chalcone-synthase (CHS) enzymes [27,28]. 

Interestingly, at T_S7_ an increase in the Flav Index on the leaf abaxial side from both the control and UV-B treatments was also observed (Supplementary Figure S1). This may indicate that other compounds absorbing in the region at 375 nm (Dualex^®^ excitation wavelength), not specifically related to UV filtering, have increased on this leaf side, possibly due to the decrease in temperature or other factors during storage. It is important to mention that non-glandular trichomes have been previously detected only in the abaxial side of basil leaves [29]. This type of trichomes, however, do not possess a secretory mechanism and can accumulate large quantities of phenolics (flavonoids and HCAs) that are non-covalently bound to the cell walls [30]. In addition, the abaxial leaf-side of basil leaves may have a high density of stomata [31], whose guard cells contain wax-bound phenolics [32,33]. All these phenolic compounds localized in the abaxial epidermal structures may play a role in protecting the leaf against different abiotic stresses and could contribute to the increment of the Flav Index on this side of basil leaves observed at T_S7_ (Supplementary Figure S1).

Considering the polyphenolic composition of leaf extracts, in accordance to previous investigations, our results showed a predominance of HCAs in the chromatographic profile, mainly rosmarinic, chicoric, and caffeic acid derivatives (Supplementary Figure S2, Figure 2) [9,34,35]. Despite previous studies having reported the presence of some flavonoids in *O. basilicum* leaves, they indicated HCAs as the most abundant compounds, including rosmarinic, caffeic, caftaric, and chicoric acids [9,34]. In addition, we found catechin and its derivatives, as previously detected by Jayasinghe et al. (2003) [34]. 

In our study, all classes of polyphenols (and also their total amount) showed higher contents in UV-B leaves with respect to the control (Figure 2). However, this increment was observed not immediately after the UV-B exposure (at T_S0_) but after the storage period (at T_S7_) (Figure 2). These results may suggest a delayed response in phenolics biosynthesis after the stimulation triggered by UV-B exposure. Indeed, the *de novo* biosynthesis after the upregulation of UVdefensive genes requires a certain amount of time [4,36]. We hypothesize that the accumulation of polyphenols observed after storage could be the result of metabolic processes induced by UV-B exposure and started before leaf sampling. Indeed, UV-B treatment seems to increase not only the transcription of *PAL* (the gene of phenylalanine ammonia-lyase [19,37]), but also directly the PAL activity [37,38,39,40]. The carbon used for polyphenol biosynthesis could derive by the release of soluble carbohydrates caused by changes in the activity of sucrose-hydrolyzing and sucrose-synthesizing enzymes as well as by higher respiratory rates, since, as has been reported in the literature, both these processes are positively affected by UV-B treatments [39,41]. In addition, it has been suggested that also the activity of cinnamate-4-hydroxylase (C4H) as well as methylation and hydroxylation reactions may be stimulated by UV-B treatments, taking around four days to generate higher amounts of *p*-coumaric, caffeic, ferulic, and sinapic acids in white cabbage leaves [40]. Therefore, the overall increase in phenolics triggered by UV-B could be detected only some period after the radiation exposure, as previously reported also in *Arabidopsis thaliana* plants [42].

These results suggest that UV-B exposure induces an initial accumulation of epidermal phenolics acting as UV-filters (as observed by the increment in the Flav Index a T_S0_, Figure 1) and, only afterwards, an increment of these compounds in the mesophyll tissue, offering a further antioxidant protection. Indeed, polyphenolic compounds inner-located in the mesophyll can protect the tissues against the action of ROS produced in response to several stresses [5,43,44]. 

This hypothesis is supported by our data on antioxidant activity (Table 1). Indeed, no differences were observed between the EC_50_ values of control and UV-B leaves at T_S0_, while a noticeably higher antioxidant activity in UV-B extracts was observed at T_S7_ compared to the controls (Table 1). The increase in antioxidant activity by exposure to UV-B in basil leaves has already been shown in the literature [3,11]. The different compounds detected in the extracts analyzed in this study, especially caffeic, rosmarinic, and chicorid acids, have been shown to possess remarkable biological and pharmacological activities both in vitro and in vivo [45,46,47], including a high antioxidant capacity. Consequently, the increase in these compounds in basil leaves may enhance their nutraceutical value. In addition, we observed a strong correlation between this activity and the amounts of polyphenols, especially with rosmarinic acid (Table 2). Although being not the most abundant substance detected, rosmarinic acid was shown to highly contribute to the antioxidant activity in basil leaves (higher values of *r* coefficient) [46]. In accordance with our findings, Castronuovo et al. (2019) [48] also found a high linear correlation between the amount of rosmarinic acid and the DPPH (2,2-diphenyl-1-picrylhydrazyl) assay results, with a coefficient *r* = 0.70, the highest value among the other compounds detected in that study [48]. This compound was the only one that showed a decrease in concentration in the control leaves after storage, and this decrease probably resulted in the lower antioxidant capacity of the control leaf extracts at T_S7_ compared to T_S0_ (Figure 2, Table 1). A decrease of 30% in rosmarinic acid content in basil leaves stored for nine days has been already observed [49], thus indicating that the storage conditions at 10 °C were detrimental only for this compound.

Another essential evaluation in the quality of leafy vegetables submitted to storage is the assessment of the chlorophyll content, which can also be affected by UV-B radiation [7]. In our study, the content of chlorophylls did not change in UV-B leaves compared to controls in any storage time (T_S0_ or T_S7_) (Figure 4), as previously observed by Mosadegh et al. (2018) [12] in *O. basilicum* leaves exposed to low doses of UV-B. 

Although the most common response to storage is the degradation of chlorophylls by the action of some enzymes, including chlorophyll oxydases [50], the increase or lack of changes in the chlorophyll content of leaves submitted to storage can also be found in the literature [7,51]. Indeed, the degradation of chlorophyll in basil detached leaves is likely to be dependent on storage temperature and seems to evidently occur only in a very late time of storage [13]. Previous investigations on fruit and vegetables have shown that UV-B treatment can suppress chlorophyll degradation during storage, thus suggesting that UV-B irradiation is a usable method for prolonging the postharvest life [52,53]. As has already been observed for other vegetables and also for basil [13,54], we observed a decrease in the carotenoid content at T_S7_ (Figure 4). In addition, we did not detect differences in the carotenoid contents between the light treatments (controlvs UV-B), thus indicating that carotenoid degradation is not induced or increased by the UV-B light treatment (Figure 4).

### 3.2. Visual Quality of O. basilicum Leaves

In order to ensure the quality of leafy-cut vegetables, not only the nutraceutical but also the visual characteristics are commonly evaluated [7,15]. Among these features, texture, determined by the degree of dehydration, and color are factors that especially influence the consumers’ choice [7,15]. 

In this study, we did not observe an effect of UV-B radiation on the general visual quality of *O. basilicum* leaves (Figure 5). Indeed, the UV-B treatment did not induce any visible necrosis, dark spots, or visible injuries in leaves at both T_S0_ and T_S7_ compared to controls (Figure 5). This is an important result, since it indicates that the doses of UV-B applied in our treatment possibly did not cause deleterious effects, such as the breakdown of essential macromolecules (DNA, lipids, and proteins) and oxidative damages, all commonly reported for UV-B exposure [2,24,55]. In fact, the leaf fresh weight decreased similarly in both treatments during the whole storage time (Table 3). The comparable values in the fresh weight loss rate found here (around 30%) were also observed in a previous study with *O. basilicum* leaves stored at 5 °C for ten days [10]. 

As already mentioned, another important visual quality characteristic to check in stored vegetables is the color. The evaluation of this feature in post-harvest quality studies comprises the analysis of values expressed in the CIELAB color scale (defined by the Commission Internationale de l’éclairage), with *L* (lightness), *a* (redness), and *b* (yellowish) as parameters from which is possible to calculate the total color changes—∆E [7,56]. Here, we observed that storage similarly increased the *L* values in leaves from both light treatments, indicating the lightening of them. Higher values in the *L* parameter have already been described for other green vegetables submitted to storage, such as green tomatoes, broccoli, rocket, Brussels sprouts, spinach, and lettuce [57,58,59,60]. Comparing the UV-B and control leaves, the treatment with radiation resulted in lower values of *L*, indicating a darker color of the UV-B leaves compared to the controls (Table 4). Previous studies have reported that the UV exposure can induce darkening [61,62]. In our study, it is likely that the UV-B has contributed to retaining the chlorophyll, thus increasing the darkness of the UV-B-treated leaves.

The higher *a* values from leaves of both light conditions indicates that they become less green, as could be expected with the storage due to the senescence processes that are commonly described [7,15]. Similar results were observed in other stored leafy vegetables, including spinach, broccoli, and lettuce [57,58]. 

Finally, the ∆E was shown to be similar at T_S7_ between the control and UVB leaves. In particular, a ∆E value higher than 3.0 indicated a very distinct difference in color from the beginning to the end of the storage period [56]. The lack of difference in this value between the UV-B and control, in addition, agrees with the similar general visual appearance and FWLR % of the leaves. All these results together indicate that UV-B application was not detrimental to the visual quality of the *O. basilicum* leaves.

### 3.3. Applicability of Dualex^®^ Instrument as a Non-Destructive Tool to Monitor the Phytochemical Quality of Detached Leaves of O. basilicum

As already stated, the Dualex^®^ device was employed in our experiment to monitor the changes in the level of leaf epidermal phenolics and chlorophylls. This sensor combines leaf transmittance and fluorescence measurements to provide chlorophyll (Chl) and flavonols (Flav) indexes, this last based on the absorption of flavones and flavonols at 375 nm [14,63]. The Dualex^®^ device allows one to measure the leaf epidermal flavonol index by an indirect method based on the detection of chlorophyll fluorescence. The comparison of this instrument with SPAD (Soil Plant Analysis Development) and other optical sensors for chlorophyll detection has been previously reported [63], while this is the first report of the measurement of the Flav Index to monitor the post-harvest quality of leafy products. 

We verified that the Dualex^®^ Flav Index is reliable with the detected content of polyphenols in *O. basilicum* leaves. A correlation analysis between the Dualex^®^ index and the HPLC-DAD quantification of polyphenols showed a high and significant correlation for all compounds, indicating that their amount and changes during storage can be easily and non-destructively inferred by this device (Table 5). Up to now, there are few studies reporting the positive correlation between the Flav Index and the amounts of flavonols [18,64,65]. Yet, to the best of our knowledge this is the first report of the correlation of the Flav Index and non-flavonoid phenolics—in this case, HCAs. This positive correlation was not previously observed [64]. In our investigation, the most abundant phenolics increased at T_S7_ in UV-B leaves were chicoric and rosmarinic acids (Figure 2). These compounds have absorption spectra with a λ_máx_ at about 350 nm, significantly extending to longer wavelengths above 375 nm. It is, therefore, reasonable to suppose that these HCAs could contribute, at least partially, to the Flav Index signal. In fact, HCAs have already been shown to decrease the epidermal transmittance at 366 nm and also at 375 nm in sunflower, even without the presence of flavonoids in this tissue [66]. 

However, we found a discrepancy between the Flav index and the HPLC-DAD quantification at T_S0_ (Figure 1, Figure 2). Indeed, while at this time the Flav Index increased in the UV-B leaves compared to the controls, the total amount of polyphenols did not show differences between the light treatments (Figure 1, Figure 2). Consequently, it is likely that also other compounds present in leaf surface tissues may have interfered with the Flav Index measurement, possibly contributing to the enhancement of this index. Indeed, *O. basilicum* leaves are rich in glandular trichomes, anatomical structures found in epidermis [29,67]. These trichomes accumulate essential oils especially rich in phenylpropanoids [9,29,67] and lipophilic flavones, mainly salvigenin and nevadensin [68,69,70]. The essential oils components and these surface flavones could also play a role in protection against UV radiation [68,69]. Although we did not evaluate the essential oil composition and its changes, the stimulus of essential oils biosynthesis by UV-B in the leaves of *O. basilicum* has already been described in the literature [71,72,73]. For instance, Ioannidis et al. (2002) [72] reported that a few days of supplemental UV-B radiation in basil plants was required to fulfill both peltate and capitate trichomes with a larger amount of essential oils with respect to the controls. The essential oils can extend their absorption up to the visible spectra range [74], and the surface lipophilic flavones have a λ_max_of up to 360 nm [69]. We believe, therefore, that these compounds may contribute to the observed increment in the Flav Index of basil leaves exposed to UV-B radiation. 

Considering the Chl Index, other studies have described, in several plant species, a linear correlation with the chlorophyll content in non-stressed leaves [63,75]. We did not observe a significant correlation between the total chlorophyll content and the Chl Index because we considered detached leaves under storage. In fact, the water loss of these leaves (Table 3) increases the light pathlength within the leaf by multiple scattering, decreasing leaf transmittance and resulting in an apparent increase in chlorophyll [76]. Similar effects of changes in leaf water content on the SPAD chlorophyll meter readings have previously been observed [77]. Indeed, we found a strong negative and significant correlation (*r* = −0.76, *p* = 0.004) between the Chl Index and the basil leaf fresh weight evaluated from T_S0_ to T_S7_, confirming the above explanation. Therefore, it is worth remembering that the possible effect of leaf dehydration must be taken into account in the monitoring of the chlorophyll content by optical sensors on leaves under storage.

## 4. Materials and Methods 

### 4.1. Plant Material 

Sixteen certified potted plants of basil (*Ocimum basilicum* L. var. Genovese, Lamiaceae) were bought, at the same moment, in a local supermarket in Florence, Italy. These plants were at similar developmental stage (around ten nodes and 0.2 m of height) and were kept under the same conditions until the beginning of the experiment in a growth chamber at the University of Florence (Department of Agriculture, Food, Environment and Forestry—DAGRI).

During the acclimation and experimental periods, the photosynthetically active radiation (PAR), measured by a Li-CoR Radiation Sensor LI-190, was 150 μmol m^−2^ s^−1^ in 8 h of photoperiod (from 9:00 to 17:00), and the temperature was 24.9 ± 0.8 °C. The plants were rotated daily in order to avoid positional effects inside the chamber, and they were watered every day (20 mL each).

### 4.2. Light Treatments, Storage Conditions and Sampling Details

**Figure 6:**
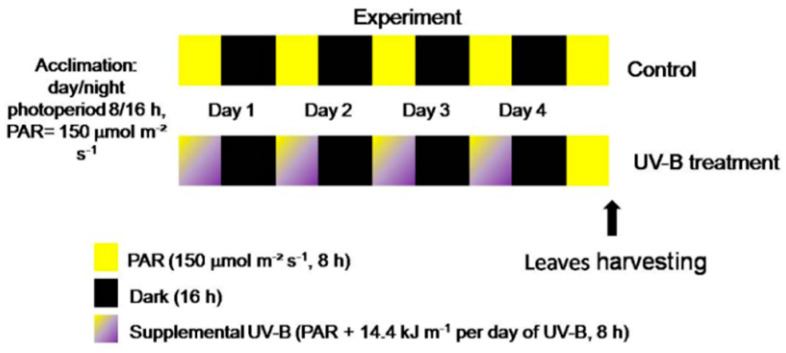
Scheme of the experimental light conditions.

The potted (0.5 L) basil plants were randomly distributed between two light treatments in the growth chamber: 8 plants were cultivated under white light (control) and the other 8 under white light supplemented with UV-B radiation (UV-B treatment) (Figure 6).

The white light was supplied by two white tubular fluorescent 40 W lamps (F40T12 Standard Phillips^®^) for both the control and UV-B treatment. The UV-B radiation was supplied by one tubular 20 W broadband UV-B lamp (290–320 nm; UV-B Medical Phillips^®^ TL UV-B G13 T12) covered with a cellulose acetate film in order to block UV-C wavelengths (<290 nm) that can possibly be emitted by the lamp. The mean irradiance of the UV-B radiation was 0.5 kg s^−3^ (0.5 W m^−2^, 8 h per day, corresponding to 14.4 kJ m^−2^ of UV-B daily dose) and was measured by a PD300-UV Ophir^®^ radiometer (with a 313 nm flat sensor, positioned horizontally during the measurements). The UV-B total daily and total doses were chosen based on previous works applying UV-B radiation to *O. basilicum* plants [11,12].

The UV-B radiation was applied during four consecutive days. The plants were kept under white light for one additional day, and then all the leaves (from the 3rd, 4th, and 5th nodes) of plants from both light treatments were harvested (Figure 6).

At the harvesting time, six leaves collected from four plants were pooled together, divided into three samples, and immediately frozen in liquid nitrogen for the biochemical analyses (corresponding to time zero of storage—”T_S0_”, *n* = 3). Other leaves were collected from other four plants (6 leaves per plant), pooled together and randomly divided into three plastic packs covered by food-quality film (8 leaves per pack, *n* = 3) and stored in fridge at 10 °C in the dark for seven days (corresponding to day seven of storage—time seven, “T_S7_”). The packing, conditions of storage, and total storage time were defined according to the maintenance instructions written in the commercial packs of basil leaves.

Phytochemical analyses (polyphenols and photosynthetic pigments), antioxidant capacity, Dualex^®^ measurements, and colorimetric analyses were performed at T_S0_ and T_S7._ In addition, the fresh weight loss rate was assessed at 0, 4, and 7 d of storage (T_S0_, T_S4_, and T_S7_).

### 4.3. Non-Destructive Analysis of Epidermal Flavonol Content (Flav Index) and Chlorophyll Content (Chl Index) by Dualex^®^

The content of epidermal phenolics (Flav Index) and of chlorophyll (Chl Index) in leaves of plants from both light conditions (control and UV-B treatment) at T_S0_ and T_S7_ were non-destructively measured by the Dualex^®^ sensor (Dualex^®^ Scientific+, Force-A, Orsay, France). It consists of a leaf-clip device, as previously described by Goulas et al. (2004) [78]. The chlorophyll content (Chl Index) is assessed as the difference in the leaf light transmission between 710 and 850 nm. The leaf epidermal UV-absorbing compounds are measured on the basis of the chlorophyll fluorescence screening method [14,78] and calculated as Flav Index = Log (ChlF_R_/ChlF_UV_), where ChlF is the emission of chlorophyll fluorescence excited in the red (R, 650 nm) or ultraviolet (UV, 375 nm). The measurements were conducted on both leaf sides, adaxial and abaxial, at three different spots per leaf. The average Flav Index was given for the adaxial epidermis, abaxial epidermis, and for both—the Total Flav Index (sum of the adaxial and abaxial Flav Index values) and the Chl Index was represented as a mean of the Chl Index values obtained with the measurements of both leaf sides.

### 4.4. Polyphenolic Analysis by HPLC-DAD

Leaf material (300 mg) sampled at T_S0_ and T_S7_ was extracted with3 × 5.0 mL ethanol 75% (pH 2.5 adjusted with formic acid) and the supernatant was partitioned with 3 × 5 mL of *n*-hexane. The extract was then reduced to dryness, and the residue was resuspended with 3.0 mL of a methanol/water solution (1:1 v/v).

Aliquots (15 μL) of the samples were injected into a Perkin^®^ Elmer Flexar liquid chromatograph equipped with a quaternary 200Q/410 pump and an LC 200 diode array detector (DAD) (all from Perkin Elmer^®^, Bradford^®^, CT, USA). The stationary phase consisted in an Agilent^®^ Zorbax^®^ SB-18 column (250 × 4.6 mm, 5 µm), kept at 30 °C. The eluents were (A) acidified water (at pH 2.5 adjusted with HCOOH) and (B) acetonitrile (at pH 2.5 adjusted with HCOOH). The following solvent gradient (v/v) was applied: 0–1 min (3% B), 1–49 min (3–40% B), 49–59 min (40% B), 59–60 min (40–3% B). The flow elution was 0.6 mL min^−1^. 

The chromatograms were obtained at 280 and 330 nm. The identification and quantification (in g kg^−1^ of dry weight) of the most abundant polyphenols were carried out based on the retention time, UV spectra and comparison with standards (gallic, rosmarinic, caffeic, chlorogenic, and chicoric acids, as well as epicatechin). Rosmarinic acid, gallic acid, and epicatechin were used to obtain calibration curves.

### 4.5. Spectrophotometric Quantification of Photosynthetic Pigments

The method of Lichtenhaler and Buschmann (2001) [79] was used for the spectrophotometric analysis of the photosynthetic pigments (chlorophylls and carotenoids). Briefly, the leaf material (150 mg) sampled at T_S0_ and T_S7_ was extracted with 2 × 1.0 mL of acetone (5 g L^−1^ CaCO_3_) in an ice bath in the dark for 15 min. After centrifugation, the absorbance of the supernatant at 470, 645, and 662 nm was measured using a PerkinElmer^®^ Lambda 25 UV/VIS spectrophotometer and acetone as blank. The content of chlorophyll *a* (Chla), chlorophyll *b* (Chlb), total chlorophyll, and carotenoids was calculated according to Lichtenhaler and Buschmann (2001) [79], using the following Equations (1)–(3):

Chl_a_ = 11.24 × A_662_ − 2.04 × A_645_, (1)

Chl_b_ = 20.13 × A_645_ − 4.19 × A_662_, (2)

Carotenoids = (1000 × A_470_ − 1.90 × C_a_ − 63.14 × C_b_)/214, (3)

where Chl_a_ is the amount of chlorophyll *a*; Chl_b_ is the amount of chlorophyll *b*; A_662_, A_645_, and A_470_ are the absorbencies at 662, 645, and 470 nm, respectively. The results were expressed in g kg^−1^ of dry weight.

### 4.6. Antioxidant Capacity of the Leaf Extracts

The same extracts obtained used for the polyphenolic analyses were measured for their free radical scavenging potential (antioxidant capacity), using the (2,2-diphenyl-1-picrylhydrazyl) DPPH assay [80]. Briefly, diluted samples of the extracts (0.5 mL) were added to 0.5 mL of DPPH solution (0.1 mM in methanol; Sigma-Aldrich^®^, Milan, Italy) and the mixture was allowed to react at room temperature for 40 min in the dark. This time was defined based on the kinetic analysis results of each extract and standards (data not shown). The absorbance was then measured at 518 nm using a PerkinElmer^®^ Lambda 25 UV/VIS spectrophotometer. The absorbance of the blank (0.5 mL methanol and 0.5 mL samples) and the negative control (0.5 mL methanol and 0.5 mL DPPH solution) were also evaluated and the analyses were conducted in triplicate. 

The percentage of antioxidant activity (AA%) and the EC_50_ (concentration of the extract to obtain 50% of the total antioxidant activity, in g L^−1^ of extract) values were calculated with the Microsoft Excel^®^ software (Microsoft corporation, New York, USA). The following equation (4) was used to calculate the AA%:

AA% = 100 − {[(ABS_sample_− ABS_blank_) × 100]/ABS_negative control_} (4)

The correlation analysis (Pearson correlation test) between the content of the detected polyphenols and the antioxidant capacity was performed, in order to evaluate which compound or class might be the main responsible for the antioxidant potential.

### 4.7. Percentage of Fresh Weight Loss Rate (% FWLR)

For the evaluation of the fresh weight loss rate, the eight pooled leaves of each pack were weighted (fresh weight—FW) in digital balance (Precisa^®^ Top PanBalance model 125A, Switzerland) at T_S0_, T_S4_, and T_S7_. The following equation was applied (5):

% FWLR = (T_S0_ FW − T_S_ FW/T_S0_ FW) × 100. (5)

### 4.8. Colorimetric Analysis

The leaf surface color was evaluated using a Portable Digital Colorimeter (SNDWAY^®^ model NH310, Beijing, China). The parameters *L*, *a*, and *b* were measured at T_S0_ and T_S7_, in three different spots of all the leaves of each pack, on adaxial surfaces, since it is the surface generally observed by the consumers. The average values of the parameters analyzed indicate the whiteness/darkness (*L*), ranging from 0 to 100 (0—black, 100—white); the redness/greenness (*a*) and the yellowness/ blueness coordinates (*b*), these last two being the chromatic components [56]. From these parameters (*L*, *a* and *b*), the total color change (∆E) was calculated and compared. This parameter indicates the magnitude of color difference after storage (T_S7_) compared to before (T_S0_). The following formula (6) was used [56]:

∆E = (∆a^2^ + ∆b^2^ + ∆L^2^)^1/2^. (6)

### 4.9. Applicability of the Non-Destructive Dualex^®^ Device

To evaluate the possible applicability of the non-destructive Dualex^®^ device to assess the leaf content of polyphenols and chlorophylls, a correlation analysis was conducted considering the samples of both light treatments (UV-B and control) and the different sampling times (T_S0_ and T_S7_) (*n* = 12). The correlation analysis was performed between the Total Flav and Chl Index values (from Dualex^®^) and the respective content of classes of polyphenols (HPLC-DAD analysis) and chlorophyll contents (spectrophotometric analysis) using the Pearson correlation test.

### 4.10. Statistical Analysis

All the data were analyzed using SigmaPlot^®^ Systat^®^ software (version 12.5, Systat Software, Inc. San Jose, CA, USA). A two-way ANOVA (factors: sampling time and light treatment) followed by Tukey post-hoc test was used. For all data, the homogeneity of variance by Levene’s test and the normality of the data by the Shapiro–Wilk test were evaluated. The detection of outliers was performed by Grubbs’ test. For all the tests, the differences were considered significant when *p* ≤ 0.05.

## 5. Conclusions

We observed that the pre-harvest UV-B treatment may be a promising tool for increasing the beneficial health components (especially hydroxycinnamic acid derivatives) and antioxidant capacity in leaves of *O. basilicum* further submitted to storage, without causing damaging effects to their visual quality. These results are important considering the nutraceutical value of the leaves, their commercial distribution, and also their possible longer maintenance.

We also highlighted the potential use of the Dualex^®^ device to reliably assess the overall polyphenolic content of basil leaves, as well as its promising use as surface sensor, giving information about the phytochemical richness in *O. basilicum* leaf epidermal surface. Further studies are needed to better understand the potential application of this tool in the assessment of essential oils contents in glandular trichomes.

## Figures and Tables

**Table 1:** Antioxidant activity (EC_50_ values in g L^−1^) of leaf extracts of *O. basilicum* at the end of the light treatment (T_S0_) and after seven days of storage (T_S7_) from plants cultivated under control (white light) and supplemental UV-B radiation.

Light Treatments	Moment of Harvesting	
	T_S0_	T_S7_
Control	0.13 ± 0.07 ^b^	0.24 ± 0.04 ^a^
UV-B	0.16 ± 0.02 ^a, ns^	0.05 ± 0.009 ^b,^***

Mean values ± SD (*n* = 3). Letters show the comparison between different times (T_S0_ vs. T_S7_) within the same light treatment. Different letters indicate significant differences between the values (*p* < 0.05), while asterisks show significant differences between light treatments at the same time of storage (*** *p* ≤ 0.001); ns = not significant.

**Table 2:** Pearson correlation analysis between the antioxidant capacity (EC_50_ values) and the polyphenolic content of leaf extracts.

Polyphenolic Content	Pearson Coefficient—*r* (EC_50_ Values)	*p* Value
Total HCAs	−0.74	0.006 **
Total polyphenols	−0.65	0.02 *
Caffeic acid	−0.49	0.10 ^ns^
Catechin derivative	−0.41	0.19 ^ns^
Chicoric acid	−0.67	0.02 *
Rosmarinic acid	−0.80	0.0003 ***

Asterisks show significant correlations among parameters (* *p* ≤ 0.05; ** *p* ≤ 0.01; *** *p* ≤ 0.001); ns = not significant (*p* > 0.05).

**Table 3:** Percentage loss in fresh weight (% FWLR) during the storage time (at T_S4_ and T_S7_) of *O. basilicum* leaves from control and supplemental UV-B light treatments.

Light Treatments	Storage Time	
	T_S4_	T_S7_
Control	20.1 ± 7.0 ^b, ns^	32.7 ± 6.8 ^a, ns^
UV-B	21.5 ± 9.2 ^b, ns^	35. 5 ± 8.5 ^a, ns^

Mean values ± SD (*n* = 3). Letters show the comparison between different storage times (T_S4_ vs. T_S7_) within the same light treatment. Different letters indicate significant differences between the values (*p* < 0.05), while asterisks show significant differences between light treatments at the same time of storage; ns = not significant.

**Table 4:** Colorimetric parameters of *O. basilicum* leaves from control and supplemental UV-B light treatments before and after the storage time (at T_S0_ and T_S7_, respectively).

Samples	Color Parameters		
	*L*	*a*	∆E
Control T_S0_	65.2± 0.6 ^b^	−4.7 ± 0.5 ^b^	-
UV-B T_S0_	64.3 ± 0.8 ^b,^*	−4.4 ± 0.5 ^b, ns^	-
Control T_S7_	66.1 ± 0.2 ^a^	−3.0 ± 0.1 ^a^	3.4 ± 0.9
UV-B T_S7_	65.4 ± 0.4 ^a,^*	−2.9 ± 0.2 ^a, ns^	2.9 ± 0.9 ^ns^

Mean values ± SD (*n* = 3). Letters show the comparison between different storage times (T_S0_ vs. T_S7_) within the same light treatment. Different letters indicate significant differences between the values (*p* < 0.05), while asterisks show the significant difference between light treatments at the same time of storage (* *p* ≤ 0.05); ns = not significant.

**Table 5:** Pearson correlation analysis between the non-destructive (Dualex^®^) and destructive contents of polyphenols (*n* = 12).

Polyphenolic Content	Pearson Coefficient—*r* (Total Flav Index)	*p* Value
Total HCAs	0.85	0.0004 ***
Total polyphenols	0.86	0.0004 ***

Asterisks show a significant correlation between the parameters (*** *p* ≤ 0.001).

## Supplementary Materials

The following are available online at https://www.mdpi.com/2223-7747/9/6/797/s1: Figure S1: Flav Index in the adaxial epidermis (a) and abaxial epidermis (b) of leaves of *O. basilicum* plants cultivated under white light and supplemental UV-B radiation extracted immediately after the light treatment (before storage T_S0_) or after seven days of storage (T_S7_); Figure S2: Representative chromatograms of *Ocimum basilicum* leaf extracts at 280 and 330 nm.

## References

[1] Tohge T., Fernie A.R. (2017). Leveraging Natural Variance towards Enhanced Understanding of Phytochemical Sunscreens. Trends Plant Sci..

[2] Neugart S., Schreiner M. (2018). UVB and UVA as eustressors in horticultural and agricultural crops. Sci. Hortic..

[3] Dou H., Niu G., Gu M. (2019). Pre-Harvest UV-B Radiation and Photosynthetic Photon Flux Density Interactively Affect Plant Photosynthesis, Growth, and Secondary Metabolites Accumulation in Basil (Ocimum Basilicum) Plants. Agronomy.

[4] Zhang W., Jiang W. (2019). UV treatment improved the quality of postharvest fruits and vegetables by inducing resistance. Trends Food Sci. Technol..

[5] Agati G., Azzarello E., Pollastri S., Tattini M. (2012). Flavonoids as antioxidants in plants: Location and functional significance. Plant Sci..

[6] Martins N., Barros L., Ferreira I.C.F.R. (2016). In vivo antioxidant activity of phenolic compounds: Facts and gaps. Trends Food Sci. Technol..

[7] Brazaitytė A., Vaštakaitė-Kairienė V., Rasiukevičiūtė N., Valiuškaitė A. (2019). UV LEDs in Postharvest Preservation and Produce Storage. Ultraviolet LED Technology for Food Applications.

[8] Toscano S., Trivellini A., Cocetta G., Bulgari R., Francini A., Romano D., Ferrante A. (2019). Effect of Preharvest Abiotic Stresses on the Accumulation of Bioactive Compounds in Horticultural Produce. Front. Plant Sci..

[9] Filip S. (2017). Basil (Ocimum basilicum L.) a Source of Valuable Phytonutrients. Int. J. Clin. Nutr. Diet..

[10] Sharma R., Bhatia S., Kaur P. (2018). Influence of packaging and storage conditions on biochemical quality and enzymatic activity in relation to shelf life enhancement of fresh basil leaf. J. Food Sci. Technol..

[11] Ghasemzadeh A., Ashkani S., Baghdadi A., Pazoki A., Jaafar H.Z.E., Rahmat A. (2016). Improvement in Flavonoids and Phenolic Acids Production and Pharmaceutical Quality of Sweet Basil (Ocimum basilicum L.) by Ultraviolet-B Irradiation. Molecules.

[12] Mosadegh H., Trivellini A., Ferrante A., Lucchesini M., Vernieri P., Mensuali A. (2018). Applications of UV-B lighting to enhance phenolic accumulation of sweet basil. Sci. Hortic..

[13] Costa L., Montano Y.M., Carrion C., Rolny N., Guiamet J.J. (2013). Application of low intensity light pulses to delay postharvest senescence of Ocimum basilicum leaves. Postharvest Biol. Technol..

[14] Julkunen-Tiitto R., Nenadis N., Neugart S., Robson M., Agati G., Vepsalainen J., Zipoli G., Nybakken L., Winkler B., Jansen M.A.K. (2014). Assessing the response of plant flavonoids to UV radiation: An overview of appropriate techniques. Phytochem. Rev..

[15] Ma L., Zhang M., Bhandari B., Gao Z. (2017). Recent developments in novel shelf life extension technologies of fresh-cut fruits and vegetables. Trends Food Sci. Technol..

[16] Li Y.F., Liu Y., Wang X., Li Y., Han R. (2019). Lower levels of UV-B light trigger the adaptive responses by inducing plant antioxidant metabolism and flavonoid biosynthesis in Medicago sativa seedlings. Funct. Plant Biol..

[17] Barnes P.W., Flint S.D., Tobler M.A., Ryel R.J. (2016). Diurnal adjustment in ultraviolet sunscreen protection is widespread among higher plants. Oecologia.

[18] Bidel L.P., Chomicki G., Bonini F., Mondolot L., Soulé J., Coumans M., La Fisca P., Baissac Y., Petit V., Loiseau A. (2015). Dynamics of flavonol accumulation in leaf tissues under different UV-B regimes in Centella asiatica (Apiaceae). Planta.

[19] Morales L.O., Tegelberg R., Brosche M., Keinänen M., Lindfors A., Aphalo P.J. (2010). Effects of solar UV-A and UV-B radiation on gene expression and phenolic accumulation in *Betula pendula* leaves. Tree Physiol..

[20] Bernula P., Crocco C.D., Arongaus A.B., Ulm R., Nagy F., Viczián A. (2017). Expression of the UVR8 photoreceptor in different tissues reveals tissue-autonomous features of UV-B signalling. Plant Cell Environ..

[21] Day T.A., Martin G., Vogelmann T.C. (1993). Penetration of UV-B radiation in foliage: Evidence that the epidermis behaves as a non-uniform filter. Plant Cell Environ..

[22] Rodríguez-Calzada T., Qian M., Strid Å., Neugart S., Schreiner M., Torres-Pacheco I., Guevara-González R.G. (2019). Effect of UV-B radiation on morphology, phenolic compound production, gene expression, and subsequent drought stress responses in chili pepper (Capsicum annuum L.). Plant Physiol. Biochem..

[23] Comont D., Winters A., Gwynn-Jones D. (2012). Acclimation and interaction between drought and elevated UV-B inA. thaliana: Differences in response over treatment, recovery and reproduction. Ecol. Evol..

[24] Robson T.M., Hartikainen S., Aphalo P.J. (2014). How does solar ultraviolet-B radiation improve drought tolerance of silver birch (B etula pendula Roth.) seedlings?. Plant Cell Environ..

[25] Tossi V.E., Regalado J.J., Iannicelli J., Laino L.E., Burrieza H.P., Escandón A.S., Pitta-Álvarez S.I. (2019). Beyond Arabidopsis: Differential UV-B Response Mediated by UVR8 in Diverse Species. Front. Plant Sci..

[26] Kaplan F., Kopka J., Sung D.Y., Zhao W., Popp M., Porat R., Guy C.L. (2007). Transcript and metabolite profiling during cold acclimation of Arabidopsis reveals an intricate relationship of cold-regulated gene expression with modifications in metabolite content. Plant J..

[27] Bilger W., Rolland M., Nybakken L. (2007). UV screening in higher plants induced by low temperature in the absence of UV-B radiation. Photochem. Photobiol. Sci..

[28] Olsen K.M., Lea U.S., Slimestad R., Verheul M., Lillo C. (2008). Differential expression of four Arabidopsis PAL genes; PAL1 and PAL2 have functional specialization in abiotic environmental-triggered flavonoid synthesis. J. Plant Physiol..

[29] Gul S., Ahmad M., Zafar M., Bahadur S., Sultana S., Ashfaq S., Ullah F., Kilic O., Hassan F.U., Siddiq Z. (2019). Foliar epidermal anatomy of Lamiaceae with special emphasis on their trichomes diversity using scanning electron microscopy. Microsc. Res. Tech..

[30] Karabourniotis G., Liakopoulos G., Nikolopoulos D., Bresta P. (2019). Protective and defensive roles of non-glandular trichomes against multiple stresses: Structure–function coordination. J. For. Res..

[31] Jensen N.B., Clausen M.R., Kjaer K.H. (2018). Spectral quality of supplemental LED grow light permanently alters stomatal functioning and chilling tolerance in basil (Ocimum basilicum L.). Sci. Hortic..

[32] Misra B.B., Acharya B.R., Granot D., Assmann S.M., Chen S. (2015). The guard cell metabolome: Functions in stomatal movement and global food security. Front. Plant Sci..

[33] Watkins J., Hechler P.J., Muday G.K. (2014). Ethylene-induced flavonol accumulation in guard cells suppresses reactive oxygen species and moderates stomatal aperture. Plant Physiol..

[34] Jayasinghe C., Gotoh N., Aoki T., Wada S. (2003). Phenolics Composition and Antioxidant Activity of Sweet Basil (Ocimum basilicumL.). J. Agric. Food Chem..

[35] Lee J., Scagel C.F. (2009). Chicoric acid found in basil (Ocimum basilicum L.) leaves. Food Chem..

[36] Jansen M.A.K., Hectors K., O’Brien N.M., Guisez Y., Potters G. (2008). Plant stress and human health: Do human consumers benefit from UV-B acclimated crops?. Plant Sci..

[37] Cantarello C., Volpe V., Azzolin C., Bertea C. (2005). Modulation of enzyme activities and expression of genes related to primary and secondary metabolism in response to UV-B stress in cucumber (*Cucumis sativus* L.). J. Plant Interact..

[38] Liu L., McClure J.W. (1995). Effects of UV-B on activities of enzymes of secondary phenolic metabolism in barley primary leaves. Physiol. Plant..

[39] Chen Z., Ma Y., Weng Y., Yang R., Gu Z., Wang P. (2019). Effects of UV-B radiation on phenolic accumulation, antioxidant activity and physiological changes in wheat (*Triticum aestivum* L.) seedlings. Food Biosci..

[40] Harbaum-Piayda B., Palani K., Schwarz K. (2016). Influence of postharvest UV-B treatment and fermentation on secondary plant compounds in white cabbage leaves. Food Chem..

[41] Interdonato R., Rosa M., Nieva C.B., González J.A., Hilal M., Prado F.E. (2011). Effects of low UV-B doses on the accumulation of UV-B absorbing compounds and total phenolics and carbohydrate metabolism in the peel of harvested lemons. Environ. Exp. Bot..

[42] Hectors K., Van Oevelen S., Geuns J., Guisez Y., Jansen M.A.K., Prinsen E. (2014). Dynamic changes in plant secondary metabolites during UV acclimation in Arabidopsis thaliana. Physiol. Plant..

[43] Rice-Evans C.A., Miller N.J., Paganga G. (1996). Structure-antioxidant activity relationships of flavonoids and phenolic acids. Free. Radic. Biol. Med..

[44] Naikoo M.I., Dar M.I., Raghib F., Jaleel H., Ahmad B., Raina A., Khan F.A., Naushin F. (2019). Role and Regulation of Plants Phenolics in Abiotic Stress Tolerance. Plant Signal. Mol..

[45] Gunia-Krzyżak A., Słoczyńska K., Popiół J., Koczurkiewicz-Adamczyk P., Marona H., Pękala E. (2018). Cinnamic acid derivatives in cosmetics: Current use and future prospects. Int. J. Cosmet. Sci..

[46] Amoah S.K.S., Sandjo L.P., Kratz J.M., Biavatti M.W. (2016). Rosmarinic Acid—Pharmaceutical and Clinical Aspects. Planta Med..

[47] Li Z., Feng H., Han L., Ding L., Shen B., Tian Y., Zhao L., Jin M., Wang Q., Qin H. (2020). Chicoric acid ameliorate inflammation and oxidative stress in Lipopolysaccharide and d-galactosamine induced acute liver injury. J. Cell. Mol. Med..

[48] Castronuovo D., Russo D., Libonati R., Faraone I., Candido V., Picuno P., Andrade P.B., Valentão P., Milella L. (2019). Influence of shading treatment on yield, morphological traits and phenolic profile of sweet basil (Ocimum basilicum L.). Sci. Hortic..

[49] Fratianni F., Cefola M., Pace B., Cozzolino R., De Giulio B., Cozzolino A., D’Acierno A., Coppola R., Logrieco A., Nazzaro F. (2017). Changes in visual quality, physiological and biochemical parameters assessed during the postharvest storage at chilling or non-chilling temperatures of three sweet basil (Ocimum basilicum L.) cultivars. Food Chem..

[50] Kaewsuksaeng S., Urano Y., Aiamla-Or S., Shigyo M., Yamauchi N. (2011). Effect of UV-B irradiation on chlorophyll-degrading enzyme activities and postharvest quality in stored lime (Citrus latifolia Tan.) fruit. Postharvest Biol. Technol..

[51] Martínez-Sánchez A., Lozano-Pastor P., Artés-Hernández F., Artés F., Aguayo E. (2019). Preharvest UV-C treatment improves the quality of spinach primary production and postharvest storage. Postharvest Biol. Technol..

[52] Srilaong V., Aiamla-Or S., Soontornwat A., Shigyo M., Yamauchi N. (2011). UV-B irradiation retards chlorophyll degradation in lime (Citrus latifolia Tan.) fruit. Postharvest Biol. Technol..

[53] Aiamla-Or S., Kaewsuksaeng S., Shigyo M., Yamauchi N. (2010). Impact of UV-B irradiation on chlorophyll degradation and chlorophyll-degrading enzyme activities in stored broccoli (Brassica oleracea L. Italica Group) florets. Food Chem..

[54] Rodriguez-Amaya D.B. (1999). Changes in carotenoids during processing and storage of foods. Arch. Latinoam. Nutr..

[55] Hideg É., Jansen M.A.K., Strid Å. (2013). UV-B exposure, ROS, and stress: Inseparable companions or loosely linked associates?. Trends Plant Sci..

[56] Pathare P., Opara U.L., Al-Said F.A.-J. (2012). Colour Measurement and Analysis in Fresh and Processed Foods: A Review. Food Bioprocess Technol..

[57] Kasım M.U., Kasım R. (2017). Yellowing of fresh-cut spinach (Spinacia oleracea L.) leaves delayed by UV-B applications. Inf. Process. Agric..

[58] Gnanasekharan V., Shewfelt R., Chinnan M. (1992). Detection of Color Changes in Green Vegetables. J. Food Sci..

[59] Koukounaras A., Siomos A.S., Sfakiotakis E. (2007). Postharvest CO_2_ and ethylene production and quality of rocket (Eruca sativa Mill.) leaves as affected by leaf age and storage temperature. Postharvest Biol. Technol..

[60] Kowalczyk D., Kordowska-Wiater M., Kałwa K., Skrzypek T., Sikora M., Łupina K. (2019). Physiological, qualitative, and microbiological changes of minimally processed Brussels sprouts in response to coating with carboxymethyl cellulose/candelilla wax emulsion. J. Food Process. Preserv..

[61] Abdipour M., Hosseinifarahi M., Naseri N. (2019). Combination method of UV-B and UV-C prevents post-harvest decay and improves organoleptic quality of peach fruit. Sci. Hortic..

[62] Scattino C., Castagna A., Neugart S., Chan H.M., Schreiner M., Crisosto C.H., Tonutti P., Ranieri A. (2014). Post-harvest UV-B irradiation induces changes of phenol contents and corresponding biosynthetic gene expression in peaches and nectarines. Food Chem..

[63] Cerovic Z.G., Masdoumier G., Ben Ghozlen N., Latouche G. (2012). A new optical leaf-clip meter for simultaneous non-destructive assessment of leaf chlorophyll and epidermal flavonoids. Physiol. Plant..

[64] Agati G., Cerovic Z.G., Marta A.D., Di Stefano V., Pinelli P., Traversi M.L., Orlandini S. (2008). Optically-assessed preformed flavonoids and susceptibility of grapevine to Plasmopara viticola under different light regimes. Funct. Plant Biol..

[65] Agati G., Tuccio L., Kusznierewicz B., Chmiel T., Bartoszek A., Kowalski A., Grzegorzewska M., Kosson R., Kaniszewski S. (2015). Nondestructive Optical Sensing of Flavonols and Chlorophyll in White Head Cabbage (Brassica oleraceaL. var.capitatasubvar.alba) Grown under Different Nitrogen Regimens. J. Agric. Food Chem..

[66] Stelzner J., Roemhild R., Garibay-Hernández A., Harbaum-Piayda B., Mock H.P., Bilger W. (2019). Hydroxycinnamic acids in sunflower leaves serve as UV-A screening pigments. Photochem. Photobiol. Sci..

[67] Gang D.R., Simon J., Lewinsohn E., Pichersky E. (2002). Peltate Glandular Trichomes of Ocimum basilicum L. (Sweet Basil) Contain High Levels of Enzymes Involved in the Biosynthesis of Phenylpropenes. J. Herbs Spices Med. Plants.

[68] Berim A., Hyatt D.C., Gang D.R. (2012). A set of regioselective O-methyltransferases gives rise to the complex pattern of methoxylated flavones in sweet basil. Plant Physiol..

[69] Grayer R., Veitch N.C., Kite G.C., Price A.M., Kokubun T. (2001). Distribution of 8-oxygenated leaf-surface flavones in the genus Ocimum. Phytochemistry.

[70] Berim A., Park J.-J., Gang D.R. (2014). Unexpected roles for ancient proteins: Flavone 8-hydroxylase in sweet basil trichomes is a Rieske-type, PAO-family oxygenase. Plant J..

[71] Chang X., Alderson P.G., Wright C.J. (2009). Enhanced UV-B radiation alters basil (*Ocimum basilicum* L.) growth and stimulates the synthesis of volatile oils. J. Hortic. For..

[72] Ioannidis D., Bonner L., Johnson C.B. (2002). UV-B is Required for Normal Development of Oil Glands in Ocimum basilicum L. (Sweet Basil). Ann. Bot..

[73] Johnson C.B., Kirby J., Naxakis G., Pearson S. (1999). Substantial UV-B-mediated induction of essential oils in sweet basil (Ocimum basilicum L.). Phytochemistry.

[74] Al-Fartosy A., Al-Rikaby A.K.J. (2007). The Antioxidative Action of Monoterpene from Loranthus europaeus L.seeds. Basrah J. Agric. Sci..

[75] Dong T., Shang J., Chen J.M., Liu J., Qian B., Ma B.L., Morrison M.J., Zhang C., Liu Y., Shi Y. (2019). Assessment of Portable Chlorophyll Meters for Measuring Crop Leaf Chlorophyll Concentration. Remote. Sens..

[76] Vogelmann T.C. (1993). Plant Tissue Optics. Annu. Rev. Plant Biol..

[77] Martinez D.E., Guiamét J.J. (2004). Distortion of the SPAD 502 chlorophyll meter readings by changes in irradiance and leaf water status. Agronomie.

[78] Goulas Y., Cerovic Z.G., Cartelat A., Moya I. (2004). Dualex: A new instrument for field measurements of epidermal ultraviolet absorbance by chlorophyll fluorescence. Appl. Opt..

[79] Lichtenthaler H.K., Buschmann C. (2001). Chlorophylls and Carotenoids: Measurement and Characterization by UV-VIS Spectroscopy. Curr. Protoc. Food Anal. Chem..

[80] Kandi S., Charles A.L., Sridhar K. (2019). Statistical comparative study between the conventional DPPH spectrophotometric and dropping DPPH analytical method without spectrophotometer: Evaluation for the advancement of antioxidant activity analysis. Food Chem..

